# Comparison of risk assessment based on clinical judgement and Cariogram in addition to patient perceived treatment need

**DOI:** 10.1186/s12903-016-0238-4

**Published:** 2016-07-07

**Authors:** Gunnel Hänsel Petersson, Sigvard Åkerman, Per-Erik Isberg, Dan Ericson

**Affiliations:** Department of Cariology, Faculty of Odontology, Malmö University, SE-205 06 Malmö, Sweden; Department of Orofacial Pain and Jaw function, Faculty of Odontology, Malmö University, Malmö, Sweden; Department of Statistics, Lund University School of Economics and Management, Lund University, Lund, Sweden

**Keywords:** Caries risk assessment, Dental caries, Guidelines, Prediction, Risk model

## Abstract

**Background:**

Predicting future risk for oral diseases, treatment need and prognosis are tasks performed daily in clinical practice. A large variety of methods have been reported, ranging from clinical judgement or “gut feeling” or even patient interviewing, to complex assessments of combinations of known risk factors. In clinical practice, there is an ongoing continuous search for less complicated and more valid tools for risk assessment. There is also a lack of knowledge how different common methods relates to one another. The aim of this study was to investigate if caries risk assessment (CRA) based on clinical judgement and the Cariogram model give similar results. In addition, to assess which factors from clinical status and history agree best with the CRA based on clinical judgement and how the patient’s own perception of future oral treatment need correspond with the sum of examiners risk score.

**Methods:**

Clinical examinations were performed on randomly selected individuals 20–89 years old living in Skåne, Sweden. In total, 451 individuals were examined, 51 % women. The clinical examination included caries detection, saliva samples and radiographic examination together with history and a questionnaire. The examiners made a risk classification and the authors made a second risk calculation according to the Cariogram.

**Results:**

For those assessed as low risk using the Cariogram 69 % also were assessed as low risk based on clinical judgement. For the other risk groups the agreement was lower. Clinical variables that significantly related to CRA based on clinical judgement were DS (decayed surfaces) and combining DS and incipient lesions, DMFT (decayed, missed, filled teeth), plaque amount, history and soft drink intake. Patients’ perception of future oral treatment need correlated to some extent with the sum of examiners risk score.

**Conclusions:**

The main finding was that CRA based on clinical judgement and the Cariogram model gave similar results for the groups that were predicted at low level of future disease, but not so well for the other groups. CRA based on clinical judgement agreed best with the number of DS plus incipient lesions.

## Background

Caries risk assessment (CRA) is aimed at identifying the risk for future disease for an individual or a group of persons [1, 2] and to be able to predict which individuals will develop cavities in the near future. A valid risk assessment would enable targeted preventive measures to be taken, that could save precious resources on costly restorative treatment procedures [3]. Most dentists perform some form of caries risk assessment based on their clinical judgement (CRA based on clinical judgement), which together with past caries experience has been shown to have good predictive power [4, 5]. It is unclear, however, how and what information dentists systematically incorporate into their treatment decisions [6, 7].

Various methods have been assessed for their predictive ability in relation to caries outcome [5] and the Cariogram, a computer based program (CRA based on the Cariogram), is one extensively studied method in which a risk profile is generated upon entering categorised clinical and history data into the program [8–12]. An ideal CRA model should be easy to use in daily practice [12]. The only validated CRA model in prospective cohort trials up to now is the Cariogram [13]. CRA based on clinical judgement is based on how the dentist, by using history and clinical data of the patient, predicts future caries development. This CRA based on clinical judgement is sometimes also called ‘gut-feeling’ [6, 14]. The Swedish Council on Technology Assessment in Health Care, concludes that the accuracy to identify risk and absence of risk in various studies or prediction models is not impressing [5]. Nevertheless, methods including clinical judgement are abundant in dentistry and well accepted by the profession [14, 15]. Many risk assessment systems, including the one used by the Swedish Public Dental Service (PDS), include a concept of risk assessment and risk grouping in order to direct the caregiver and act as a helping tool to plan an optimal care. However, for caries, such programs have not been compared to validated models for CRA as the Cariogram has been. It is therefore of interest to compare the outcome of caries risk determined by clinical judgement and the Cariogram which has been studied in relation to predictive power [8, 9, 16].

Another important issue is that it is not clearly understood which factors clinicians base their risk assessment on. There are reasons to believe that obvious clinical findings as large caries lesions, large amount of plaque or severe oral dryness are apparent findings to the clinicians to assess a high risk. This topic needs to be further investigated [13].

The patients self-perceived oral health is associated with caries and prosthetic status [17], therefore it is of interest to compare clinical findings and self-perception in this study. It is thus also of interest to investigate how risk assessment based on clinical judgement expressed as the sum of examiners risk score i.e. of caries, periodontal, general and technical risks and patients perception of future dental treatment need are related.

Our hypotheses were:

- CRA based on clinical judgement and CRA based on the Cariogram give a fair agreement since some of the variables, such as caries prevalence and incidence are common for both models.
- factors like caries experience and dental plaque from the clinical status and history agree best with CRA based on clinical judgement.
- the patient’s own perception of future oral treatment need do not correspond with the sum of examiners risk score based on clinical judgement.

The aims of this study were to investigate:

- if CRA based on clinical judgement using guidelines and CRA based on the Cariogram model give similar results and if risk profiles varies with age.
- which factors from the clinical status and history, evident to the examiner, agree best with the CRA based on clinical judgement.
- if the patient’s own perception of future oral treatment need correspond with the sum of examiners risk score based on clinical judgement.

## Methods

### Study group

A sample of 1000 individuals, 20–89 years old registered as living in the county of Skåne, Sweden, was randomly selected for a larger cross sectional study of oral health [18]. From the original sample, a total of 451 individuals (47 %), 232 women (51 %) and 219 men (49 %) agreed to participate and were examined clinically. Data from 446–450 subjects could be analysed for different purposes as full data from the questionnaire, the history and the clinical examination were missing for one to five individuals. The study design was approved by the Ethical Board at the University of Lund, Sweden (Dnr. 513/2006). Patients were informed about the survey and signed a written consent.

Information from the questionnaire, history, radiological and clinical findings [18] relevant to this study, relating to caries and patients self-assessment of future oral treatment need were extracted for further analyses. Variables evaluated by clinical examiners in this study were: past and new caries experience (DMFT, DS, incipient lesions), general diseases or conditions and medication associated with dental caries, plaque amount, oral dryness, intake frequency of soft drinks and dental erosions.

### Questionnaire

Before the clinical examinations the participants answered a questionnaire containing questions concerning oral health and oral health related factors [19]. The non-response analysis has been described earlier [18]. Individuals in the age group 80–89 were less likely to participate (OR = 2.82). Patients scored their own future treatment need in five grades from ‘very low’ to ‘very high’ as well as ‘don’t know’ by answering the question: How do you judge your future oral treatment need? Due to few individuals (*n* = 19) in the ‘very high risk’ group, they were included in the ‘high risk’ group.

### History

General diseases, conditions and medication associated with dental caries was recorded and entered into the Cariogram according to the manual. Estimations of intake frequency of soft drinks, citrus fruits and apples were recorded in the history, and graded; ‘less than once a week, once a week, daily and several times a day’.

### Clinical examination

The clinical examinations were performed during 2007–2008 and took place at the Faculty of Odontology at Malmö University, Sweden, and at three clinics at the PDS in the county of Skåne. The examinations were performed by eight dentists all employed at the Department of Oral Diagnostics, Faculty of Odontology, Malmö University and 90.5 % were performed by four of them. The examiners were coordinated regarding the diagnostic criteria through comprehensive written instructions, practice and through discussing clinical cases before the clinical examinations. All patients were examined using a standard examination procedure in standard surgeries [18].

Decayed, missing and filled teeth and surfaces (DMFT and DS including incipient lesions) were recorded. Caries lesions were determined using standard clinical criteria aided by mirror, probe (Hu-Friedy EXD57) and digital bite-wing radiographs. On radiographs, lesions that only included the enamel were recorded as incipient lesions, and lesions that extended into the dentine were recorded as manifest caries. Clinically, incipient lesion criterion was opacity with or without roughness. Activity was determined by appearance of lesions and active dentine caries was determined if the surface was soft by the probe [18].

Dental erosions were assessed on buccal and lingual surfaces of upper incisors and canines using a scoring system (grade 0–4) where the severity of the erosion was graded according to Johansson and co-workers [20]. Dental erosion was included in the study as some risk factors overlap with those for caries and presence of erosions might influence self-assessment of oral health. Plaque was recorded as present or not on four surfaces on each tooth. The total percentage of plaque covered surfaces was calculated for each patient.

Oral dryness was determined by the examiner by the use of a mouth mirror at the clinical examination. The score ‘Severe’ was recorded if the mouth mirror adhered to the buccal mucosa. ‘Some dryness’ was scored if there was a friction between the mouth mirror and the buccal mucosa.

Paraffin-stimulated whole saliva was collected for 5 min for estimation of secretion rate and expressed as ml/min. Salivary mutans streptococci, lactobacilli and saliva buffering capacity were determined with Dentocult® SM - Strip mutans, Dentocult® LB and Dentobuff® Strip, respectively. Test kits were obtained from Orion Diagnostica, Espoo, Finland and handled according to the instructions of the manufacturer.

### CRA based on clinical judgement

The clinical guidelines for risk assessment were based on patient’s history, past and present disease, general and technical conditions. The caries risk assessment was based on past and present caries, clinical activity of lesions, and evaluation of risk factors (dietary content and intake frequency of carbohydrates, fluoride exposition and plaque amount, general health, medication, social situation and expected cooperation). The periodontal risk assessment was based on presence of gingivitis, marginal bone loss, plaque situation, general health, medication, social situation and expected cooperation. The general risk assessment was based on presence of general diseases, medication, dental anxiety and communication ability. The technical conditions were based on the extent of restorations and prosthetic reconstructions incorporating general health, medication, social situation and expected cooperation. The clinical guidelines for risk assessment were modified after the Public Dental Service guidelines for risk assessment in adults in Skåne region as described in Hänsel Petersson et al. 2013 [21]. The examiners had thus a reasonably good overview of the patient’s oral situation. The risk assessment was performed directly after the clinical examination and patients were scored from low to very high risk based on the protocol paired with their own clinical judgement. Caries, periodontal, technical and general risk was scored ‘low risk’ as 1 point, ‘moderate risk’ as 2, ‘high risk’ as 3 and ‘very high risk’ as 4 points in each group. To be considered as “moderate risk” indicates that the patient was categorized in between low or high risk group. Patients who are at “moderate risk” for dental caries have an uncertain probability to develop new caries lesions unlike a patient being classified to ”high risk” or “low risk”. The patients often have a combination of different underlying risk factors. The sum of examiners risk score was defined as: ‘low risk’ 4 points, ‘moderate risk’ 5–8 points and ‘high/very high risk’ 9–16 points. At this time, the dentists were not aware of the data from the questionnaire, the results of the salivary tests or the Cariogram score.

### CRA based on the Cariogram

In this study, data from the questionnaire, history, laboratory and caries data were evaluated and entered into the Cariogram model, except for dietary content where only the lactobacillus score were used [8]. The Cariogram was used in standard setting without altering the “clinical judgement”. Information included scoring of caries experience, DMFT, DS and combining DS and incipient lesions; general disease, conditions and medications associated with dental caries; dietary content based on lactobacillus test count; dietary intake frequency based on estimation of number of meals and snacks per day; plaque amount; mutans streptococci colonization using the Strip mutans test; fluoride programme based on fluoride exposure; saliva secretion using paraffin-stimulated secretion rate; saliva buffering capacity using the Dentobuff test. The intake frequency of diet, soft drinks and fruits was compiled from self-reports.

### Statistical methods

Eleven variables from the questionnaire and history, related to caries and patients self-assessment of future treatment need were extracted for further analyses.

The Pearson Chi-2 test was used to test the relation between risk assessment based on clinical judgement and CRA using the Cariogram. The same test was used to test the relation between patient’s own perceptions of future oral treatment need with the sum of examiners risk score based on clinical judgement.

The relation between CRA (scored as 0 = low-moderate and 1 = high) and clinical variables were analysed using a logistic regression. A significance level of 5 % was used in all tests. Statistical calculations were performed in the Statistical Package for the Social Science (SPSS for Windows, version 21, Chicago Ill., USA).

## Results

The relation between CRA based on clinical judgement using guidelines for risk assessment and, the assessments made by using a computerized risk assessment program, the Cariogram, is presented in Table 1. For those assessed as low risk using the Cariogram, 69.3 % also were assessed as low risk based on clinical judgement. For those assessed as moderate/high risk using the Cariogram, the agreement was lower with the CRA based on clinical judgement.

**Table 1:** Relation between CRA based on clinical judgement and CRA based on the Cariogram

	Cariogram score %- chance of avoiding caries, *n* (%)			
CRA based on clinical judgement	81–100 %	41–80 %	1–40 %	Total
	(Low risk)	(Moderate risk)	(High risk)	
Low risk	70 (69.3)	119 (42.8)	16 (23.2)	205 (45.8)
Moderate risk	26 (25.7)	103 (37.1)	22 (31.9)	151 (33.7)
High risk	5 (5.0)	56 (20.1)	31 (44.9)	92 (20.5)
Total	101	278	69	448

Chi-square *p* < 0.001

Low vs Moderate *p* = 0,011; Low vs High *p* = 0,001; Moderate vs High *p* = 0,102

**Fig. 1:**
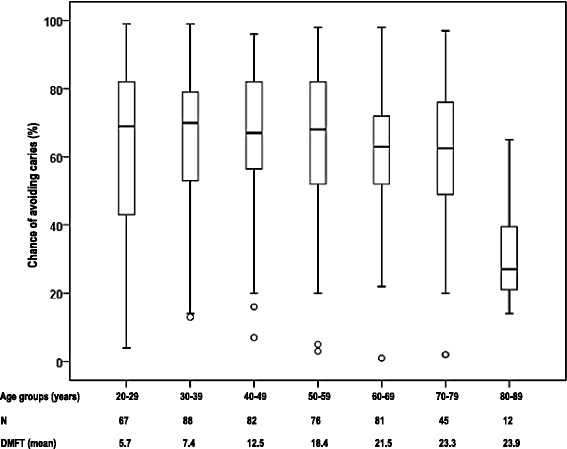
The boxplot shows the median percent chance of avoiding caries according to the Cariogram, in different age groups

Figure 1 presents the risk profile, expressed by the Cariogram, as the median ‘%-chance of avoiding caries’, in the different age groups. The median value is rather similar throughout the age groups varying between 62.5–70 %chance of avoiding caries except for the oldest group (80–89 years), where there is a lower median value (27 % chance of avoiding caries).

**Fig. 2:**
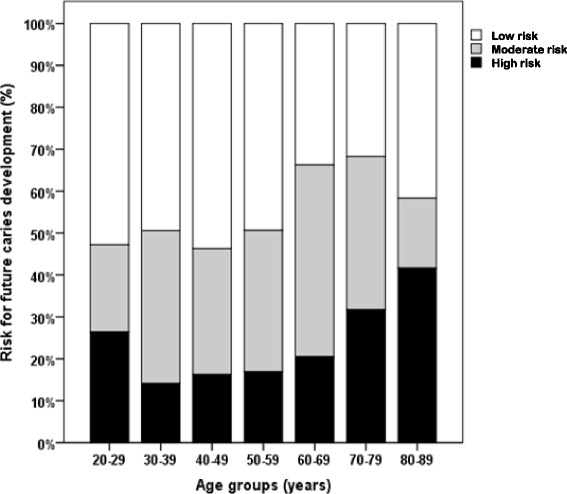
The diagram shows the distribution of risk for future caries development based on clinical judgement in different age groups

Based on clinical judgement, the risk for future caries development related to age is demonstrated in Fig. 2. In the youngest age group (20–29 years), the high risk percentage is 26 % and this high risk precentage decreases to 14 % in the age group 30–39 years and thereafter a steady increase is seen with age. In the 80–89 year age group, the high risk percentage is 42 %.

The univariate relations between clinical parameters, recorded by the examiner performing the risk assessment, and the CRA based on clinical judgement by the same examiner, are shown in Table 2. The two clinical variables which coincided best were, decayed tooth surfaces (DS) and combining DS and incipient lesions. But also DMFT value, plaque amount, and history were significantly correlated with the CRA based on clinical judgement. If the subjects had decayed tooth surfaces, the estimated risk by the examiners increased nearly four times (OR 3.96).

**Table 2:** Relations between CRA based on clinical judgement and clinical variables (logistic regression - univariate analyses), recorded by the examiner performing the risk assessment

Clinical variables	Category	*n*	OR	Lower CI	Upper CI	*P*
DMFT		450	1.07	1.04	1.11	< 0.001
DS		450	3.96	2.95	5.32	< 0.001
DS and incipient lesions		450	1.51	1.35	1.68	< 0.001
Plaque amount (%)		450	1.02	1.01	1.03	0.001
	None	404				
Oral dryness	Some + severe	45	1.89	0.96	3.72	0.076
	None	310				
Erosion score	Grade 1	100	1.42	0.83	2.42	0.449
	Grade 2 + 3 + 4	39	1.10	0.48	2.51	
History		450	1.19	1.07	1.34	0.002
	Less than once a week	192				
Intake frequency of soft drinks	Once a week	141	0.69	0.38	1.23	0.046
	Daily	96	1.31	0.73	2.34	
	Several times a day	20	2.62	1.00	6.84	
	Less than once a week	84				
Intake frequency of citrus fruits and apples	Once a week	169	1.03	0.52	2.04	0.247
	Daily	160	1.24	0.63	2.44	
	Several times a day	36	2.30	0.95	5.60	

The relation between the patient’s own perception of future oral treatment need and the sum of the examiners risk assessments based on clinical judgement of caries, periodontal, technical and general risk, is demonstrated in Table 3. The correlation between the examiners and the patient’s own assessment was significant. However, 8.5 % reported that they did not know their own future oral treatment need. 43.5 % of the subject’s self-assessment of future oral treatment need belonged to the ‘moderate’ risk group.

**Table 3:** Relation between the patient’s own perceptions of future oral treatment need with the risk assessment based on the sum of examiners risk score

Patient’s own perception of future oral treatment need (%)					
Sum of examiners risk score^a^	Minor	Moderate	Large	Do not know	Total
Low risk	16 (25.4)	29 (46.0)	13 (20.6)	5 (7.9)	63 (14.1)
Moderate risk	26 (10.6)	116 (47.2)	85 (34.5)	19 (7.7)	246 (55.2)
High risk	12 (8.7)	49 (35.8)	62 (45.2)	14 (10.2)	137 (30.7)
Total	54 (12.1)	194 (43.5)	160 (35.9)	38 (8.5)	446

Chi-square p = 0.002

^a^Refers to the summarized risk assessment based on clinical judgement of the caries, periodontal, technical and general risk conditions

## Discussion

The concept of risk based caries management is based on the assumption that dentists can use clinical indicators to classify caries risk status to predict future caries experience and thereby categorize caries risk levels [2, 22, 23]. As a consequence, interventions should be targeted to the identified risk factor(s) in each case followed by a relevant recall plan. In comprehensive clinical practice, risk factors based on past caries experience, general health, diet including intake frequency and oral hygiene are often subjectively and intuitively merged into one of several risk categories [24, 25], albeit the quality of evidence for this process is limited [26].

One aim in this study was to study the relation between CRA based on clinical judgement using clinical indicators and a software program, the Cariogram [10]. The main finding in this study was that the Cariogram estimation of the chance of avoiding caries correlated well with the clinical risk assessment for the group that were predicted at low level of future disease, but not so well for the other groups (Table 1). Thus, the present study brings novel information on the relation between use of clinical judgement for risk grouping and the computerized risk assessment program, the Cariogram.

The median risk profile, expressed by the Cariogram showing the median %-chance of avoiding caries, did not vary between the different age categories except for the oldest age group (80–89 years), where there was a significant lower median value (Fig. 1). The result is quite consistent with a report from the Social Insurance Agency in Sweden and the number of restorations, placed due to caries, correlates well with the risk assessment except for the youngest age group [27].

Similar results are shown in a recent study, were the performance of CRA in young adults using PDS guidelines was compared to the Cariogram model. The agreement was acceptable (77.5 %) for those assessed as low risk, while discrepancies were disclosed among those classified with higher risks [11].

The outcome described above is in line with previous knowledge. In most studies, the specificity is higher than the sensitivity, indicating that CRA models actually are more effective in selecting patients at low risk than those with high caries risk. All prediction models are influenced by the prevalence of disease and in a low disease prevalence population the positive predictive value is strongly influenced. In a systematic review, from the Swedish Council on Technology Assessment in Health Care, it was concluded that it was possible to identify those with a low risk of developing caries and that the current methods for risk assessment have a low accuracy [5]. This inverted way to use CRA seems however to be adopted by few [28], most reports are still focused on finding the true high-risk individuals. A significant proportion of patients who regularly attend general dental practice have repeated examinations without any need for treatment. Thus, it would be desirable to screen out these patients to concentrate the resources on those with greatest need in order to enable targeted preventive measures to be taken.

Few studies have addressed what information dentists use for the CRA and how they judge the weight of different factors. Riley and co-workers [24] tested several hypotheses related to CRA and individualized caries prevention. They showed that a substantial percentage of the dentists in this study do not perform CRA, and there is not a strong linkage between its use and use of individualized preventive regimens for adult patients. According to a questionnaire by Riley et al. [24] risk assessment of children was carried out by 76 % of all dentists but only 14 % took the advantage of structured models or protocols. For adults, 69 % of the dentists performed any kind of CRA [25]. Little is however known on the accuracy of this ‘intuitive’ way of risk assessment more than it is merely based on the past caries situation [14]. However, published protocols or guidelines seem seldom evaluated.

Another aim was thus to find out which factors from the clinical status and the history evident to the examiner that coincided best with the CRA based on clinical judgement (Table 2). The two most evident factors for the examiner were DS and combining DS and incipient lesions. This is in accordance with other studies and a systematic review [5, 14]. Most reviews of the literature on CRA have concluded that ‘past caries experience’, and especially existing active lesions, is the most powerful single predictor of future caries development at practically all ages [29–31].

In the present study, the odds ratios for plaque amount (OR 1.02) and history (OR 1.19) were small, even though they were significantly correlated with CRA based on clinical judgement (Table 2). The low impact of plaque amount for caries risk is in concordance with the ecological plaque hypothesis as well as the high impact of intake frequency of soft drinks (‘several times a day’ OR 2.62) [32]. The somewhat higher odds ratio for history seems reasonable since this parameter includes caries related diseases, medication and general health conditions. Thus, the findings of this study corroborate earlier findings on the impact of previous caries experience on the outcome of risk assessment. However, from a disease management perspective this is a less desirable outcome, considering the fact that the disease is actually manifested before it can be accurately predicted as the ultimate goal of caries management is to prevent even the earliest enamel lesions [2].

In recent years several studies have assessed self-perceived oral health and a number of clinical factors have been found to be associated with self-perceived oral health. In one recent study with the aim to explore the relative contributions of clinical variables assessing caries, periodontal and prosthetic status to self-perceived oral health among a study population ranging from 35–74 years of age, it was concluded that the presence of decayed and filled teeth were strongly associated with self-perceived oral health [33]. Self-assessed data is also suggested to be used for screening purposes for oral health service planning and for priority allocation in large adult populations [34].

Subsequently, a third aim was to investigate the relation between the patient’s own perception of future oral treatment need and the sum of examiners risk score based on clinical judgement. It was evident that individuals rating themselves to have large future oral treatment need were in agreement with the examiners rating the risk high. For the other risk groups, there was less agreement (Table 3).

Alas, around 9 % did not know their own future oral treatment need while 44 % of the subjects belong to the moderate group of self-assessment of future oral treatment need. Since the major part of the population were clustered in this moderate group or did not know about their needs, the statistical significance of the correlation to the examiners’ assessment should be interpreted with great caution. It does not necessarily infer that patients would agree with examiners on the future treatment need or risk profile, if the population was divided in more than three risk groups.

The data from this study are cross-sectional and no data on future oral disease development is available. Therefore, the results must be interpreted with caution and it would be interesting to be able to follow up the validity of the prediction models used in this study. The clinical implication for this study is that we need to improve methods for caries prediction that have a high sensitivity. The validated computerized model, the Cariogram and CRA based on clinical judgement agree acceptably for the low risk group and high specificity has been validated for the Cariogram [16].

## Conclusions

The main finding was that the agreement between CRA based on clinical judgement and the Cariogram model was highly significant for the groups with low risk. The CRA based on clinical judgement agreed with DS and combining DS and incipient lesions. Patients own perception of large treatment need agreed with the examiner’s rating.

## Abbreviations

CRA, caries risk assessment; DMFT, decayed, missed, filled teeth; DS, decayed surfaces
